# An investigation of parafoveal masks with the incremental boundary paradigm

**DOI:** 10.1371/journal.pone.0203013

**Published:** 2019-02-28

**Authors:** Florian Hutzler, Sarah Schuster, Christina Marx, Stefan Hawelka

**Affiliations:** Paris-Lodron-University of Salzburg, Department of Psychology, Centre for Cognitive Neuroscience, Salzburg, Austria; Aix-Marseille Université, FRANCE

## Abstract

Most of what we know about parafoveal preprocessing during reading is based on the boundary paradigm in combination with parafoveal masks as a presumably neutral baseline condition. Recent evidence questions the neutrality of the baseline condition by showing that parafoveal masks inflict preview costs. Using a novel, incremental boundary paradigm we studied the effect of parafoveal masks. Manipulating the salience of parafoveal previews, we found that increasing salience of the masks resulted in increasingly longer fixation times on target words, but also on pretarget words—suggesting preview costs. We conclude that the hidden preview costs of parafoveal masks in the classical boundary paradigm inflate the processing times for the baseline condition and hence lead to an overestimation of the preview benefit. Thus, the present study questions the validity of some of the conclusions drawn on the basis of the classical boundary paradigm.

## Introduction

During natural reading, we process written words with great speed and fluency. This efficient processing depends on the fact that we do not only process the word which we are currently fixating, but also preprocess the upcoming, parafoveal word. When information on the upcoming word is masked, reading speed decreases substantially [1].

### Parafoveal preprocessing and the boundary paradigm

Most of what we know about parafoveal preprocessing is based on evidence from eye movement studies. In most of these studies, parafoveal preprocessing is investigated by means of a gaze-contingent display change technique, that is, the *boundary paradigm* [2]. The boundary paradigm makes it possible to experimentally manipulate the characteristics of the preview of the upcoming, parafoveal word. To illustrate, in a sentence an invisible boundary is placed prior to a theoretically relevant target word. As long as the reader fixates to the left of the boundary, an experimentally manipulated preview is provided. When the reader’s eyes cross the boundary, the preview is replaced by the target word (see [3][4] on the question of binocular foveation and the boundary technique).

For the objective of the present study, two variants of the boundary paradigm must be distinguished. These two variants differ with respect to the *type* of preview which is provided parafoveally. This difference is theoretically relevant, because it determines, whether a baseline condition is necessary for interpretation. In a first variant, researchers explicitly investigate, whether an experimentally manipulated preview *affects* the subsequent processing of the target word. More specifically, this variant is used to explore whether a specific type of information (e.g., semantic or phonological) is extracted from the parafovea. For example, in a study on semantic preview benefit a parafoveal preview was either semantically related to a target word (e.g., preview: *puppies*; target: *kittens*) or it was semantically unrelated (e.g., *offices*; [5]). For the interpretation of this experimental design, the semantically related condition is compared to the semantically unrelated condition. In so doing, Rayner [5] did not find evidence for a semantic preview benefit (subsequent studies corroborated this finding—but see [6] [7] [8]).

The second variant of the boundary paradigm is the central one for the present study. In this variant, the parafoveal preview is (as in the variant described above) different from the target word. The crucial difference between the two variants is that—instead of an existing word—a string of letters is used to cover the target word. In the following, we will refer to this kind of preview as parafoveal *mask*. Parafoveal masking is used to estimate the magnitude of the preview benefit, that is, the extent to which readers benefit from parafoveal information. In these experiments, a parafoveal preview is presented which is either valid, that is, identical to the target word (e.g., *viewer*—*viewer*), or partially valid, for example, *vievcnr* which is an orthographically similar nonword (sharing the first 3 letters of the target word). For the interpretation of this kind of experimentation, the condition with a (partially) valid preview is compared to a baseline condition in which participants do not have a valid preview (e.g., *nmovcn*—*viewer*; see [9]). Of interest is whether and to what extent participants’ processing times (e.g., gaze durations) are shorter on the target word when they were presented with a (partially) valid preview compared to the baseline condition (i.e., the parafoveally masked preview condition).

To summarize, there are two different variants of the boundary paradigm. A baseline-condition is necessary for interpreting the results of the latter variant–which is central for the present study. Most of what we know about parafoveal preprocessing stems from experiments which applied parafoveal masks. These findings concern, for example, the magnitude of the preview benefit–which is estimated to be around 30–50 ms [9], the evidence that information of the initial letters of the parafoveal word is conducive for subsequent word recognition [10] [11] [12] [13] [14] [15] and the evidence that the parafoveal preview benefit is dependent on the precision of lexical representations [16].

### The requirement of an appropriate baseline

What requirements must an appropriate baseline condition meet? Critical is that a baseline must be neutral with respect to the subsequent process of foveal word recognition. If processing times in an experimental condition are shorter than in the baseline condition, one speaks of facilitation. If, to the contrary, processing times are longer than in the baseline condition, one speaks of inhibition or interference of the experimental condition. However, to interpret any differences between an experimental condition and the baseline condition, a parafoveal mask itself must not facilitate or interfere with the subsequent foveal word recognition. As Rayner and Slowiaczek [17] put it, “*the direction of these preview effects is crucially dependent on the choice of the baseline condition*” (*p*. 645), because “*the pattern of results changes differentially depending on which baseline we choose”* (*p*. 647).

Assume, for the purpose of illustration, a hypothetical experimental condition in which the parafoveal preview does not provide a preview benefit. In an ideal setting (i.e., when the baseline condition is indeed neutral) one would not observe any processing advantage of the experimental condition compared to the baseline condition. Thus, one could correctly assume that there is no discernible effect of the preview. However, if the baseline condition actually interfered with subsequent foveal word recognition, then the processing time for the baseline condition would be (artificially) elevated. Comparing the processing time in the experimental condition with such a non-neutral baseline condition would feign reduced processing times in the experimental condition. As a consequence, one would erroneously infer a preview benefit.

To summarize, an ideal (i.e., neutral) baseline condition neither interferes with nor facilitates the subsequent process of foveal word recognition. A neutral baseline is the prerequisite for a valid interpretation of the mask-based application of the boundary paradigm. In the following, evidence and theoretical considerations on the question of an appropriate parafoveal mask are reviewed. Thereafter, we refer to recent evidence which raises doubt about the neutrality of parafoveal masks.

### Theoretical considerations and evidence on the appropriate baseline condition

Parafoveal masking was introduced by the seminal study of Rayner, McConkie and Ehrlich [16]. Immediately afterwards, a (short-lived) theoretical discussion ensued about the right choice of baseline. The discussion, however, ceased unresolved. Researchers in the field of eye movement research on reading continued to use parafoveal masks and the neutrality of parafoveal masks was implicitly assumed, but never again explicitly investigated. In the following, we recapitulate the findings of Rayner et al. [18] and the following theoretical discourse. Going back in time 40 years is necessary, because the theoretical foundation of parafoveal masking builds on these early studies.

#### Rayner, McConkie and Ehrlich [18] and the start of a theoretical discourse

Rayner et al. [18] presented different types of parafoveal previews in order to explore which information is obtained from the parafoveal stimulus, and what effect (inhibitory or facilitatory) this information has on foveal word recognition. To investigate the potential costs and benefits of parafoveal previews, the processing times (indexed by naming latencies) in the different preview conditions were compared to a “baseline” condition in which the parafoveal preview consisted of a single asterisk. In comparison to this baseline condition, all previews elicited shorter naming latencies during the subsequent processing of the target words. Consequently, Rayner et al. [18] concluded that all previews, which were considered in the study, facilitated foveal word recognition (in various degrees). However, the debate about the choice of the “right”baseline that followed Rayner et al.’s study ([17] [19] [20]) reveals that the baseline issue was controversial and results can vary and indeed did vary (see [17]) depending on the choice of baseline. In a final comment, McClelland and O’Regan [20] agreed with Rayner and Slowiaczek [17] regarding the requirement for (but not regarding the concrete choice of) a baseline condition: „*some kind of neutral preview must be found to assess costs and benefits of information extracted from parafoveal vision*”(*p*. 653).

The boundary paradigm (using parafoveal masking) became one of the standard paradigms in reading research, despite the unresolved question which mask truly is an appropriate baseline. Today, we still lack an empirical validation of the appropriate baseline condition but researchers, it seems, chose to abide by the presupposition that their parafoveal masks are neutral. Thus, a great deal of what we know about parafoveal preprocessing is based on the boundary paradigm with parafoveal masks. This accumulated evidence, however, is possibly not based on a firm footing ([21] [22] [23]). Jordan and colleagues [24], for example, pointedly commented that “[…] *in the absence of clear unequivocal evidence that a primary experimental manipulation does not produce secondary*, *unwanted influences*, *it is prudent for researchers to seek to minimize the potential for these experimental side effects*.” (*p*. 901). In this study we will demonstrate how an empirical exploration of the costs of parafoveal masks may be possible.

#### Empirical approach for baseline testing: Within-condition baseline by incremental priming

The issue of an adequate baseline condition is not limited to parafoveal masking, but is a problem inherent to the use of baseline conditions in other domains as well [25]. Thus, a solution initially developed for another domain might also be suitable to address the issue in the boundary paradigm. Below, we will describe the incremental priming technique [26]. It was originally developed to provide a solution for the baseline problem in priming studies on visual word recognition. The rationale, however, may be also suitable for the boundary paradigm, that is, for exploring the effect (i.e., neutral, facilitatory or interfering) of parafoveal masks.

The rationale of the incremental priming technique is as follows: The informational value (i.e., the *salience*) of the primes in an experimental condition is gradually increased. The critical aspect then is how the processing times of the targets change in response to the increasing salience of the prime. If increasing salience leads to faster response times, then the prime is facilitatory. Conversely, if increasing salience leads to slower response times, then the prime interfered with the processing of the targets. Thus, the incremental priming technique provides a within-condition baseline which renders an explicit baseline condition unnecessary [26].

It is noteworthy that the findings of Rayner et al. [18] can be “re-interpreted” in terms of incremental priming. This is because Rayner and colleagues administered an additional, critical manipulation: The distance between the fixation location and the parafoveal preview was experimentally varied between 1°, 3°, and 5° of visual angle. This eccentricity manipulation is also a variation of the salience of the parafoveal preview. The closer a preview is to the fixation, the more information does it provide due to higher visual acuity. In Rayner et al., increasing fixation-preview proximity led to faster naming latencies of the target words for most preview types. Thus, a re-interpretation of the study in terms of incremental priming would attest the previews a facilitatory effect.

#### Recent evidence

Only recently, the issue of an appropriate baseline in eye movement studies using the boundary paradigm and parafoveal masks received renewed attention. Kliegl and colleagues [22] re-analysed data from a published study [27] which administered the boundary paradigm with random letter strings as masks and applied the rationale of the incremental priming technique. The authors *post-hoc* quantified the salience of the previews by analysing gaze durations on the target words for instances in which the preceding fixation was either near or remote from the target word (similar to the experimental manipulation of Rayner and colleagues [18]). The rationale is that during near fixations the parafoveal preview has a higher perceptibility (i.e., a higher salience) than during remote fixations. The findings showed that the random letter masks elicited elevated gaze durations when the previous fixation was close to the parafoveal mask compared to remote fixations—evidence that random letter masks inflict preview costs. In [21], we investigated the effects of parafoveal X-masks with the boundary paradigm and the concurrent recording of *fixation-related brain potentials* (*FRP*s [28]). We found a significantly belated occurrence of a well-established marker-effect in the condition which presented X-masks compared to a condition which did not present a parafoveal preview. In [23], we examined the effect of parafoveal X-masks and of same-shape different letter masks with the incremental boundary paradigm in children. Both types of parafoveal masks inflicted preview costs. Thus, these recent studies concurred that preview effects, which were up to now subsumed under the umbrella term *preview benefit*, are a “*complex mixture of benefits and costs*” ([22]; *p*. 596).

### Aims and outline of the present study

The central idea of the present study is that the rationale of the incremental priming technique can be applied to the boundary paradigm (henceforth: *incremental boundary paradigm*). The salience manipulation of parafoveal information enables the assessment of the true nature (i.e., facilitatory or interfering) of parafoveal masks. In Experiment 1, we apply a salience-by-distance manipulation as in Rayner et al. [18] to examine the effects of same-shape, different letter masks. Experiment 2 will introduce the method of visual degradation to realize a salience-by-perceptibility manipulation which is the prerequisite for the incremental boundary paradigm. An orthogonal combination of the salience-by-perceptibility and the salience-by-distance manipulation in Experiment 3 will allow us to determine the extent of visual degradation which is necessary to realize a preview manipulation which, on the one hand, does not result in a preview benefit and, on the other hand, does not inflict preview costs. Finally, in Experiment 4 we will apply the incremental boundary paradigm during sentence reading. Doing so will allow us to investigate i.) whether parafoveal masks inflict preview costs during natural reading and ii.) whether the incremental boundary paradigm is applicable for studying parafoveal preprocessing.

## Experiment 1: Salience-by-distance

Rayner et al.’s study [18] is the empirical foundation of the boundary paradigm. Interpreted in terms of saliency-by-distance, Rayner et al.’s findings indicated that parafoveal mask do not interfere with foveal word recognition. However, one must concede that over the last 30 years, the available technology for eye movement research has markedly improved. Moreover, the total of three participants in [18] would nowadays be considered as too few. The restricted stimulus set, with numerous repetitions of the same words, could also be considered as potentially problematic (see [19]). The aim of Experiment 1 is to reassess the effect of parafoveal masking by means of contemporary technology, a larger number of participants and a larger stimulus set. We chose the–nowadays–most commonly used parafoveal mask, that is, the same-shape, different letter mask (henceforth SSDL mask).

### Method

#### Participants

Thirtysix undergraduate native German-speaking students (26 female; mean age: 26 y, SD = 3.16, all of full age) with normal or corrected to normal vision were recruited for the experiment (because we counterbalanced the assignment of the stimuli to the experimental conditions according to a Latin-square design, the number of participants was necessarily a multiple of 6, see Material for details). The participants were naïve to the purpose of the experiments. None of the participants took part in more than one experiment of the present study. All experiments of the present study were conducted in accordance with the Code of Ethics of the World Medical Association (Declaration of Helsinki), participants gave verbal informed consent and the experiments were approved by the local ethics committee of the University of Salzburg (“Ethikkommission der Universität Salzburg”) and the data was anonymized.

#### Apparatus

Eye movements of the right eye were recorded with an EyeLink CL eye-tracker (SR-Research, CA) with a sampling rate 1 kHz. The participant’s head was stabilized by a chin-rest and a forehead-rest 52 cm in front of an LCD monitor (24 inch, 16:9 aspect ratio, resolution 1366x768). The refresh rate of the monitor was 144 Hz.

#### Material

In this experiment, we orthogonally combined the factors type of preview (two levels: valid previews vs. SSDL masks) and eccentricity (i.e., salience-by-distance, three levels: 1°, 3° and 5° to the right of fixation), resulting in a total of 6 different experimental conditions. Experimental stimuli were 360 5-letter words (exclusively nouns) which were assigned to 6 different lists (*n* = 60 each). The lists were rigorously matched on a total of 8 word characteristics (i. frequency, ii. Coltheart’ s N, iii. frequency of the highest-frequent neighbor, iv. number of higher frequency neighbors, v. frequency of the initial bigram, vi. summed bigram frequency, vii. mean bigram frequency, viii. number of syllables) and the onset phonemes of the words. Statistical comparison did not reveal differences between the lists for any of these characteristics (all *F*s < 1.34). Furthermore, we counterbalanced–according to a Latin-square design–the assignment of these lists to the experimental conditions in such a way that each list was used equally often for each of the experimental conditions (i.e., we had 6 different experimental sequences and each of these sequence was administered to 6 participants). 120 additional stimuli were used as centrally presented filler items.

#### Procedure

The experiment was programmed with the Experiment Builder software (SR-Research, CA). A horizontal 3-point calibration routine preceded the experiment and was repeated whenever the fixation check at the beginning of a trial failed. The criterion for successful calibration was a tracking error of less than 0.3° of visual angle. A trial started with the presentation of two vertical lines at the center of the screen (“fixation bars”). If the eye tracking system did not register a fixation at the fixation cross (within 500 ms), then the system was recalibrated. If the system registered a fixation on the fixation cross (minimum duration: 100 ms), stimuli were presented either at 1°, 3° or 5° to the right of the fixation (distance from middle of the fixation bars to the first letter of the stimulus) or centrally (i.e., centered at the point of fixation). The parafoveal stimulus (with a single character subtending approximately 0.3° of visual angle) was either a SSDL mask or a valid preview of the word. Display changes were realized with the boundary paradigm [2]. The invisible boundary was placed between the fixation bars and the parafoveal stimulus (located 12 pixels right to the fixation bars). Crossing the boundary triggered the display change, that is, the replacement of the mask with the target word. The centrally presented stimuli were theoretically irrelevant filler items. The aim of these filler items was to prevent anticipatory eye movements. Participants were required to name the target words as fast as possible. The naming latencies were recorded with a voice key. The experimenter terminated the trial with a button press. Correct versus incorrect pronunciations of the target words were distinguished by the experimenter by pressing different buttons.

#### Data treatment and analyses

The raw data for all Experiments is available at the Open Science Framework at osf.io/f69x8/. Naming onset latencies were measured from the onset of the first fixation on the stimulus (more specifically, in a window covering the target word and 3 characters to the left and right of the target word) after crossing the invisible boundary. Trials in which the participants undershot or overshot the stimulus plus/minus these margins were omitted (< 2% of the data). Only correctly named target words were analyzed (data omission < 0.5%). Naming latencies shorter than 200 ms or greater than 1,200 ms were considered as outliers (7% of the data).

### Results

Word lists were, as described above, rotated between the 6 conditions in such a way that each target word was presented equally often (i.e., 6 times) in each experimental condition. Rigorous counterbalancing of stimuli across participants and across conditions guarantees that mean differences between conditions are not affected by any difference that might exist between the lists [29]. Thus, “when the lists are counterbalanced over different groups of subjects, there is no need to compute (*min*)*F*’ and the simple subject analysis (averaging over items) will be correct” ([29]; p. 426). Furthermore, this approach does not only render an item-based (i.e., F_2_) analysis unnecessary. Rather, an item-based analysis of such a complete design is inherently impossible, because of missing data (each stimulus was presented 6 times in a specific condition, i.e., item-based means would be calculated on the basis of 6 trials). This approach to deal with the fixed-effect fallacy [30] was applied in each experiment and hence we performed ANOVAs over participants (i.e., F_1_) throughout the present study.

**Fig 1:**
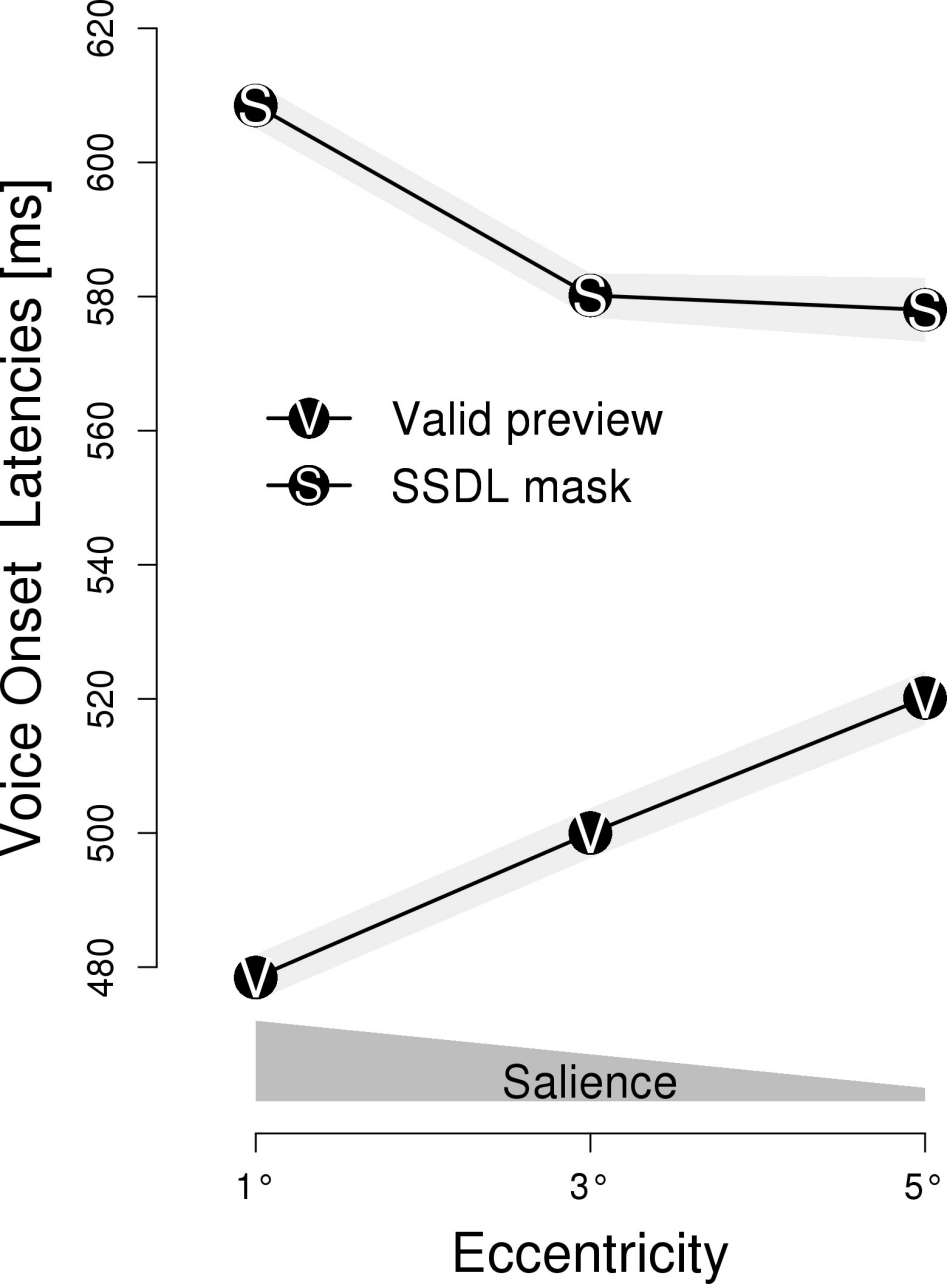
Experiment 1: Mean voice onset latency. Voice onset latencies are depicted in relation to three different eccentricities and are plotted separately for valid previews and same-shape, different letter (SSDL) masks. Polygons represent 1 SEM. The dark gray polygon above the x-axis schematically indicates the level of salience, i.e., the salience is high if the preview is close to the fixation, but decreases when the preview is farther away from the fixation.

Fig 1 depicts the naming latencies for the different types of parafoveal previews and for the different eccentricities at which these previews were presented (i.e., salience-by-distance). Critically, a 2 x 3 repeated measures ANOVA with *type of preview* (valid previews vs. masks) and *salience* (eccentricity: 1°, 3° and 5°) revealed a significant interaction between these factors; *F*(2, 70) = 71.81, *p* < .001, η = 0.018; which qualified the main effect of type of preview; *F*(1, 35) = 283.86, *p* < .001, η = 0.141; and the main effect of salience; *F*(2, 70) = 3.24, *p* < .05, η = 0.001. In Fig 1, the two theoretically relevant effects of *type of preview* are evident. First, the preview of an SSDL mask elicited longer latencies than valid previews. Second, salience had opposite effects for the SSDL masks and the valid previews. Separate ANOVAs revealed that–for valid previews–higher salience resulted in shorter naming latencies, *F*(2, 70) = 42.24, *p* < .001, η = 0.023. In contrast, for SSDL masks higher salience resulted in prolonged naming latencies, *F*(2, 70) = 24.15, *p* < .001, η = 0.016.

### Discussion

Experiment 1 aimed at reassessing the finding of Rayner et al. [18] which indicated a facilitatory effect of parafoveal masks. We interpret the findings of our re-assessment in terms of a salience-by-distance effect. The closer a parafoveal preview is to the fixation location, the higher is its salience [22]. Critical for the interpretation is how naming latencies change in response to increasing salience, that is, the slope of latencies in relation to the eccentricity. An upward slope towards the left margin of Fig 1 (i.e., prolonged latencies with increasing salience) indicates that the preview elicited processing costs. A downward slope to the left, by contrast, reveals processing benefits. Expectedly, valid parafoveal previews resulted in a preview benefit. SSDL masks induced preview costs. This finding is in accordance with the evidence for random letter masks [22].

In the following, we will argue that the logic of the incremental priming paradigm (i.e., the within-condition baseline) can be adopted to the application of parafoveal masks by systematically manipulating the salience of parafoveal previews. An experimental manipulation of eccentricity of the parafoveal preview, however, is not practicable in natural reading. If we want to develop an incremental boundary paradigm for natural reading, we must find a salience manipulation that can be realized during reading of sentences. In Experiment 2, we will assess the effects of a salience manipulation which is accomplished by systematically degrading the visual integrity of parafoveal information. We refer to this manipulation of the previews as salience-by-perceptibility manipulation.

## Experiment 2: Salience-by-perceptibility

**Fig 2:**
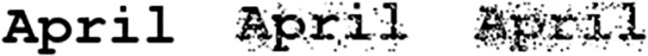
Experiment 2: Magnified depiction of visual degradation. Visual degradation was realized by pixel displacement. From left to right, the figure shows examples of 0%, 10% and 20% of pixel displacements, that is, parafoveal previews with high to low salience.

In Experiment 2 we examine the feasibility of a salience-by-perceptibility manipulation. Such a manipulation was already successfully applied in incremental *priming* studies (e.g., [26] [31]). More specifically, SSDL masks and valid previews will be visually degraded by displacing a certain amount of pixels in the bitmap of the parafoveal stimulus (see Fig 2).

### Method

#### Participants

We recruited 36 undergraduate native German-speaking students (24 female; mean age: 24 *y*, *SD* = 2.43, all of full age) with normal or corrected to normal vision (counterbalancing using a Latin-square design necessitated that the number of participants is a multiple of 6).

#### Apparatus

The eye tracker and the setup was the same as in Experiment 1.

#### Material

In this Experiment, we orthogonally combined the factors type of preview (two levels: valid previews vs. SSDL masks) and visual degradation (three levels: 0%, 10%, 20%), resulting in 6 different conditions. Stimuli were the same as in Experiment 1 and they were again Latin-square counterbalanced across conditions (i.e., 6 different experimental sequences, each administered to 6 participants).

#### Procedure

The eye tracking system was calibrated as described in Experiment 1. Again each trial began with centrally presented fixation bars and registration of the initial fixation position was as described in Experiment 1. Stimuli were presented either 2.2° to the right of the fixation or centrally. The stimuli, which were presented to the right of fixation, are the experimental stimuli (¾ of the stimuli); the centrally presented stimuli were theoretically irrelevant filler items which were not included in the analyses. The aim of these filler items was to prevent anticipatory eye movements which might have occurred, if all the stimuli were presented at the same (parafoveal) location. Of the experimental stimuli, half were parafoveally masked by an SSDL mask. The salience-by-perceptibility manipulation of the parafoveal previews (i.e., valid previews and SSDL masks) was realized by displacing either 20%, 10% or none of the black pixels of the bitmapped image of the stimuli (see Fig 2). Thus, there were 6 categories of experimental stimuli (i.e., 2 types of previews x 3 levels of salience) with *n* = 60 per category. For the display changes, the boundary paradigm was applied as in the previous experiment.

#### Data treatment and analysis

As in Experiment 1. Erroneous naming accounted for 3.6% of the data. As in Experiment 1, these trials were excluded from the analysis.

### Results

**Fig 3:**
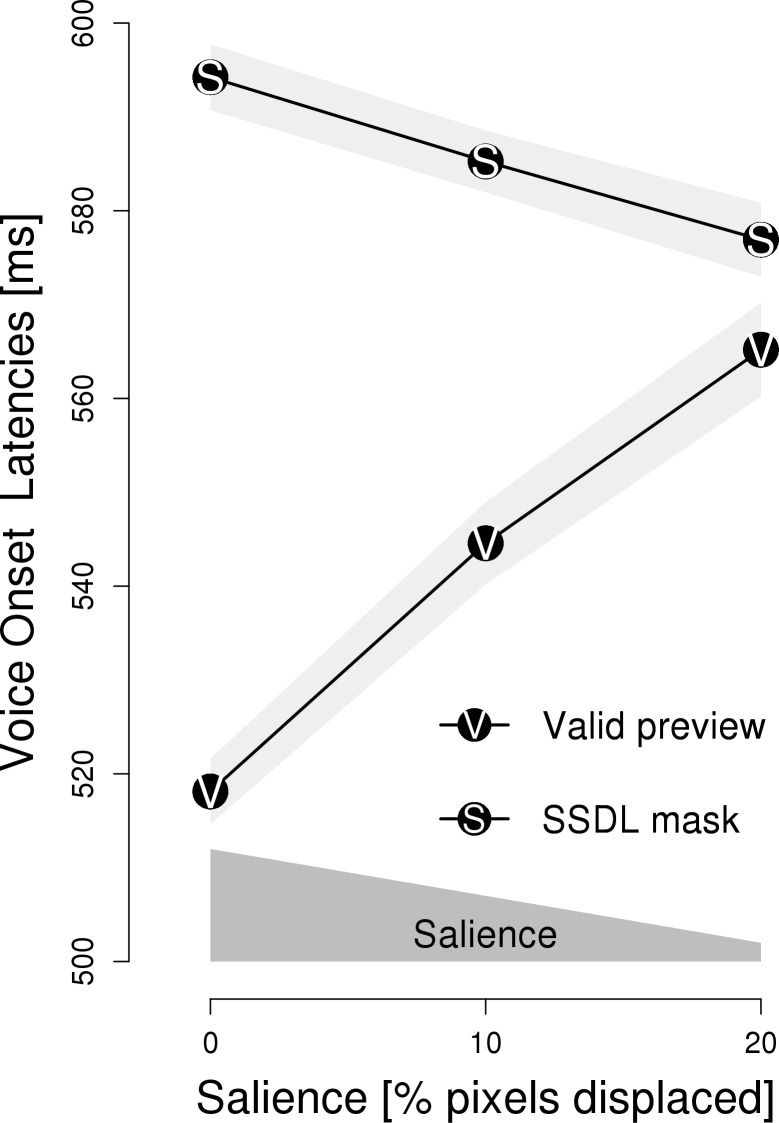
Experiment 2: Mean voice onset latency. Voice onset latencies are depicted in relation to the three levels of salience-by-perceptibility and is plotted separately for valid previews and same-shape, different letter (SSDL) masks. The light-gray areas represent 1 SEM. The dark gray polygon above the x-axis indicates the level of salience, i.e., the salience is high if the preview is visually intact (i.e., 0% of the pixels displaced), but decreases when the preview is visually degraded (i.e., when an increasing percentage of pixels is displaced).

Fig 3 depicts naming latencies in relation to different levels of visual degradation for valid previews and SSDL masks. As detailed in Experiment 1, ANOVAs were performed only over participants (i.e., F_1_), because rigorous counterbalancing by means of a Latin-square design rendered item-based analyses unnecessary and uncalled for. A 2 x 3 repeated measures ANOVA with *type of preview* (valid previews vs. masks) and salience (0%, 10% and 20% pixels displaced) revealed a significant interaction between type of preview and salience; *F*(2, 70) = 31.89, *p* < .001, η = 0.026; qualifying a main effect of preview; *F*(1, 35) = 105.67, *p* < .001, η = 0.066; and a main effect of salience; *F*(2, 70) = 7.80, *p* < .001, η = 0.006. In Fig 3, again the two theoretically relevant effects of *type of preview* are evident. First, the preview of an SSDL mask elicited longer naming latencies than valid previews. Second, salience-by-perceptibility had an opposite effect on SSDL masks and valid previews. Separate ANOVAs for each type of preview revealed that, for valid previews, higher salience resulted in shorter naming latencies–indicating preview benefits, *F*(2, 70) = 28.67, *p* < .001, η = 0.052. In contrast, for SSDL masks, higher salience resulted in prolonged naming latencies–indicating preview costs, *F*(2, 70) = 6.68, *p* < .01, η = 0.008.

### Discussion

Manipulating the salience of parafoveal previews by means of visual degradation affected naming latencies in Experiment 2. Higher salience of valid previews resulted in shorter naming latencies–reflecting a preview benefit. Higher salience of SSDL masks, by contrast, resulted in prolonged naming latencies. This finding further corroborates the conclusion from Experiment 1 that SSDL masks inflict preview costs.

In order to maximise the experimental and statistical sensitivity of the *incremental boundary approach* it is important to choose the salience manipulation in such a way that it covers the continuum from full parafoveal perceptibility (i.e., maximum preview benefit) to such a low level of salience which provides minimal parafoveal information. A low level of salience is expected to minimize preview benefit without inflicting preview costs—in the following, we label such a condition a zero-information condition. Thus, in the next experiment we investigated to what extent the perceptibility of parafoveal previews must be diminished in order to realize such a zero-information condition.

## Experiment 3: Orthogonal combination of salience-by-distance and salience-by-perceptibility

An orthogonal combination of salience-by-perceptibility with salience-by-distance will allow us to assess how different levels of visual degradation affect parafoveal preprocessing. By means of presenting visually degraded previews at different eccentricities we can extend the logic of the within-condition baseline in order to investigate the exact nature of degraded previews. As long as different eccentricities affect processing times for a certain level of degradation, parafoveal information can still be extracted from the stimulus–indicating that the extent of visual degradation did not reach a point of zero-information extraction. If, to the contrary, different eccentricities do not affect processing times, then the parafoveal preview is visually degraded to a degree which does not allow the readers to extract (facilitatory) parafoveal information. In Experiment 3, we therefore will orthogonally combine eccentricity and perceptibility for *valid previews*.

### Methods

#### Participants

We recruited 24 undergraduate native German-speaking students (12 female; mean age: 23 *y*; *SD* = 2.86, all of full age) with normal or corrected to normal vision (counterbalancing using a Latin-square design necessitated number of participants to be a multiple of 12).

#### Apparatus

Eye movements were recorded (monocular for the right eye) as in the previous experiments with the difference that participants sat at a viewing distance–held constant by a forehead and a chin rest–of 52 cm to a 17.7 inches CRT computer screen (640 x 480 pixel resolution, 45 pixels per inch, 200Hz frame rate).

#### Material

The orthogonal combination of four different levels of visual degradation (i.e., salience-by-perceptibility manipulation) and three different eccentricities (i.e., salience-by-distance manipulation) resulted in a total of 12 experimental conditions. Visual degradation was realized as in Experiment 2 (i.e., displacement of pixels) and ranged from high salience (0% pixels displaced) to low salience (30% pixels displaced) in increments of 10%. Salience-by-distance was operationalized by presenting parafoveal previews 1.5°, 3.0° or 4.5° to the right of central fixation. Experimental stimuli were 480 (5-letter) words (exclusively nouns) which were assigned to 12 different lists (*n* = 40 each). The items of each list were, as in the previous experiments, rigorously matched on several word characteristics (all *F*s <1). The presentation of the lists were Latin-square counterbalanced in such a way that each list was used equally often for each of the 12 conditions (i.e., each of the 12 different experimental sequences was administered to two participants). The words were typed in monospaced, bold font. A single character had a width of approx. 0.3° of visual angle.

#### Procedure

The eye tracking system was calibrated as described in Experiment 1, calibration was repeated at the halftime of the experimental run. Each trial started with a centrally presented fixation cross and registration of the initial fixation location was as described in Experiment 1. The invisible boundary was located 12 pixels right to the central fixation cross and parafoveal preview was manipulated in one of the four above mentioned salience levels. When the eyes crossed the boundary, the degraded version of the stimuli was replaced with their non-degraded version. Again, participants were instructed to name the words and voice onset latencies were recorded as in the previous experiments.

#### Data treatment and analysis

As in Experiment 1.

### Results

**Fig 4:**
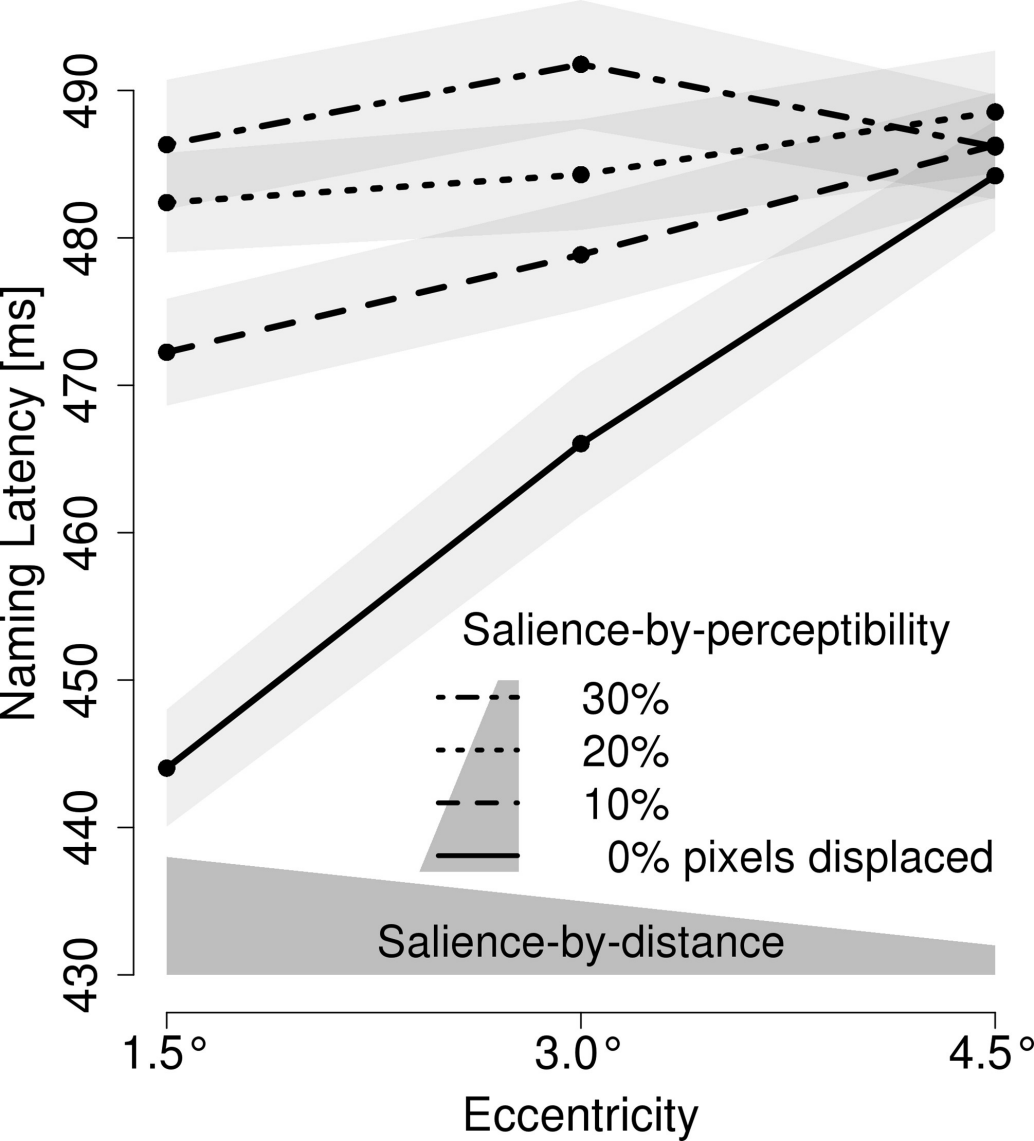
Experiment 3: Mean voice onset latency. Mean voice onset latencies are depicted in relation to the *salience* of the preview which was manipulated i.) by eccentricity, that is, the distance from fixation and ii.) by gradually diminishing the visual integrity (i.e., the perceptibility) of the parafoveal preview. The gray polygon above the x-axis illustrates the decreasing salience of the preview with increasing eccentricity. The polygon in the legend illustrates the salience of the preview in relation to its visual integrity. The light-gray areas represent 1 SEM.

Fig 4 presents the results of the orthogonal manipulation of eccentricity and visual degradation. A 3 x 4 repeated measures ANOVA with salience-by-distance (eccentricities of 1.5°, 3.0° and 4.5°) and salience-by-perceptibility (0%, 10%, 20% and 30% of the pixels displaced) revealed that an interaction of the two factors; *F*(6, 138) = 5.65, η = 0.015; qualified the main effects of salience-by-distance; *F*(2, 46) = 12.6, η = 0.014; and salience-by-perceptibility; *F*(3, 69) = 15.1, η = 0.030; all *p*s < .001.

Separate ANOVAs for the four levels of visual degradation revealed the following: For non-degraded previews, smaller eccentricities resulted in shorter naming latencies; *F*(2, 46) = 25.9, *p* < .001, η = 0.073; indicating a preview benefit. This effect was less pronounced, but significant, for the previews in the 10% degradation condition; *F*(2, 46) = 3.66, *p* < .05, η = 0.013. For the previews with 20% and 30% degradation, the analyses did not reveal effects of eccentricity, *F*s < 1. Naming latencies differed substantially between the four levels of degradation, when the stimuli were presented at the closest eccentricity; *F*(3, 69) = 22.7, *p* < .001, η = 0.096; but did not differ when the differently degraded stimuli were presented at the most remote eccentricity; *F* < 1.

### Discussion

By orthogonally combining salience-by-distance and salience-by-perceptibility, we aimed to investigate from which amount of visual degradation onwards it is no more possible to extract information from the parafoveal stimulus. Technically speaking, we aimed to investigate which amount of visual degradation constitutes a zero-information condition. Our reasoning is as follows: As long as information can be extracted from a visually degraded, parafoveal stimulus, the availability of this very information is also dependent on the eccentricity at which the parafoveal stimulus is presented (i.e., dependent on salience-by-distance). In other words, as long as a certain level of visual degradation is still susceptible to the salience-by-distance manipulation, this very level of visual degradation still allows the extraction of parafoveal information. If, however, a certain level of visual degradation is not affected by the salience-by-distance manipulation, a zero-information condition is achieved. In this experiment, we examined the effect of eccentricity (i.e., salience-by-distance) separately for the four levels of visual degradation (0%, 10%, 20% and 30% of pixels displaced) and found the expected pattern of results. For non-degraded stimuli, close proximity to the central fixation resulted in substantially faster naming latencies compared to more distant presentations. With increasing visual degradation, however, eccentricity had less impact on naming latencies: Naming latencies of stimuli degraded by 10% were less affected by eccentricity than undegraded stimuli. Naming latencies of stimuli degraded by 20% or 30% were not affected by eccentricity at all. Thus, a salience-by-perceptibility manipulation of 20% or more indeed constitutes a zero-information condition. A further methodologically relevant implication is that the visual degradation beyond the point of zero-information did not inflict preview costs. For previews degraded by 20% or 30%, there was no evidence for slower naming latencies at closer distance (which would indicate preview costs).

Based on the finding of the current experiment, for the implementation of the incremental boundary paradigm during natural reading in Experiment 4, we foresee that a pixel displacement of about 20% will be sufficient to realize a zero-information preview condition (Pixel displacements by 20% proved reliable to realize zero-information baseline for the *present* experimental setting–whether this extent of visual distortion is sufficient for stimuli of, e.g., different length or familiarity or from orthographies other than German warrants further investigation). Realizing a zero-information condition offers an additional possibility for interpreting the incremental boundary paradigm. Although the mere slope of processing times resulting from a gradual variation of salience is sufficient to differentiate preview costs from preview benefits, comparing a valid preview condition to a zero-information preview condition makes it possible to estimate the *absolute* extent of the preview benefit.

## Experiment 4: Sentence reading

Experiment 2 showed that visual degradation is a feasible manipulation of the salience of a parafoveal preview which allows us to assess preview costs and benefits. In Experiment 4, we will now implement the incremental boundary paradigm during sentence reading to address two questions. First, we will explore whether the interference of parafoveal masks (proven to exist for isolated words in Experiments 1 & 2) generalizes to sentence reading (*cf*. [22]). Second, we will investigate whether the incremental boundary paradigm allows us to reliably assess the beneficial effect of valid previews. Furthermore, this paradigm does not only make possible to analyse preview benefits and costs; it also offers the possibility to examine parafoveal-on-foveal effects (that is, the influence of parafoveal masks on the fixation times on the pretarget word). Furthermore, besides the analysis of the experimentally varied visual perceptibility of the preview, the natural variation of gaze durations on the *pretarget* word and the natural variation of the length of the incoming saccade towards the target word will allow us to analyze–post-hoc–the effects of preview time (i.e., the gaze duration on the pretarget word as proxy for the exposure duration to the parafoveal preview) and launch-site distance (i.e., salience-by-distance)–similar to the analysis of [22].

### Methods

#### Participants

Thirtytwo participants took part in Experiment 4 (counterbalancing using a Latin-square design necessitated number of participants to be a multiple of 8). They were undergraduate, native German-speaking students (21 female; mean age: 23 *y*, *SD* = 1.8, all of full age) with normal or corrected to normal vision.

#### Apparatus

Eye movements were recorded (from the right eye) as in the previous experiments. Stimuli were presented on a 17 inch CRT monitor (1024x768 pixel resolution) with a refresh rate of 150 Hz.

#### Material

In this experiment, we orthogonally combined type of preview (two levels: valid previews vs SSDL masks) and the salience of the parafoveal previews (four levels: 0%, 7%, 14% or 21% of pixels displacement—maximum displacement of 21% was chosen on the basis of the findings of Experiment 3 which revealed that a visual degradation of 20% is sufficient to realize a condition in which it is no more possible to extract information from the parafoveal stimulus)–resulting in a total of 8 experimental conditions. Experimental stimuli were 320 5-letter target words that were assigned to 8 different lists (*n* = 40 each) which were, as in the previous experiments, rigorously matched on relevant characteristics. The target words were embedded in sentences (one target word per sentence). The sentences were constructed in such a way that at least 2 words preceded (*M* = 3.03, *SD* = 1.20) and at least 2 words succeeded the target word (*M* = 4.38 , *SD* = 1.56). The sentence length ranged from 5 to 11 words (*M* = 7.41, *SD* = 1.39). The sentences were presented in a bold, monospaced font type (black on white background). Viewing distance was 52 cm. A single character subtended approximately 0.3° of visual angle. We counterbalanced–according to a Latin-square design–the assignment of the lists of sentences to the experimental conditions in such a way that each of the 8 lists of sentences was used equally often for each of the 8 experimental conditions (i.e., each sequence was presented to 4 participants; one participant, however, had to be excluded from the analysis because of technical problems).

#### Procedure

The eye tracking system was calibrated with a horizontal three-point routine. The calibration was conducted before and after the presentation of 8 familiarization trials and was repeated whenever the fixation check at the beginning of a trial failed. The criterion for successful calibration was a tracking error of less than 0.3° of visual angle. A trial started with a fixation check, that is, the presentation of a vertically centred fixation cross at the left side of the screen. Display changes were realized with the boundary paradigm [2]. The boundary was located 6 pixels (i.e., half a character) right to the pretarget word. Before the eyes crossed the boundary, the parafoveal previews were either valid previews or SSDL masks and were degraded by either 0%, 7%, 14% or 21%. When the eyes crossed the boundary, the degraded preview was replaced with the non-degraded target word. Participants were instructed to read silently for comprehension. Performance on the interspersed comprehension questions, presented orally after about every 9th sentence, was close to ceiling (*M* > 99%).

### Results

In a first analysis, we investigated the effects of masks and valid previews during the processing of the *pretarget word* (i.e., while participants fixated the word prior to the invisible boundary and while the preview–either masked or valid–is still in the parafovea). Thereafter, we present the effects of the preview manipulations *after* crossing the invisible boundary, that is, during the foveal processing of the *target word* (formerly masked and/or degraded or presented as a valid preview). Gaze durations on the pretarget and target words were submitted to separate 2 x 4 repeated measures ANOVAs with preview type (masks vs. valid previews) and salience (0%, 7%, 14%, or 21% of visual degradation) as within-subject factors. As detailed in Experiment 1, ANOVAs were performed over participants (i.e., F_1_), because rigorous counterbalancing by means of a Latin-square design rendered item-based analyses unnecessary and uncalled for.

#### Pretarget words—The effects of visual degradation of the target word

**Fig 5:**
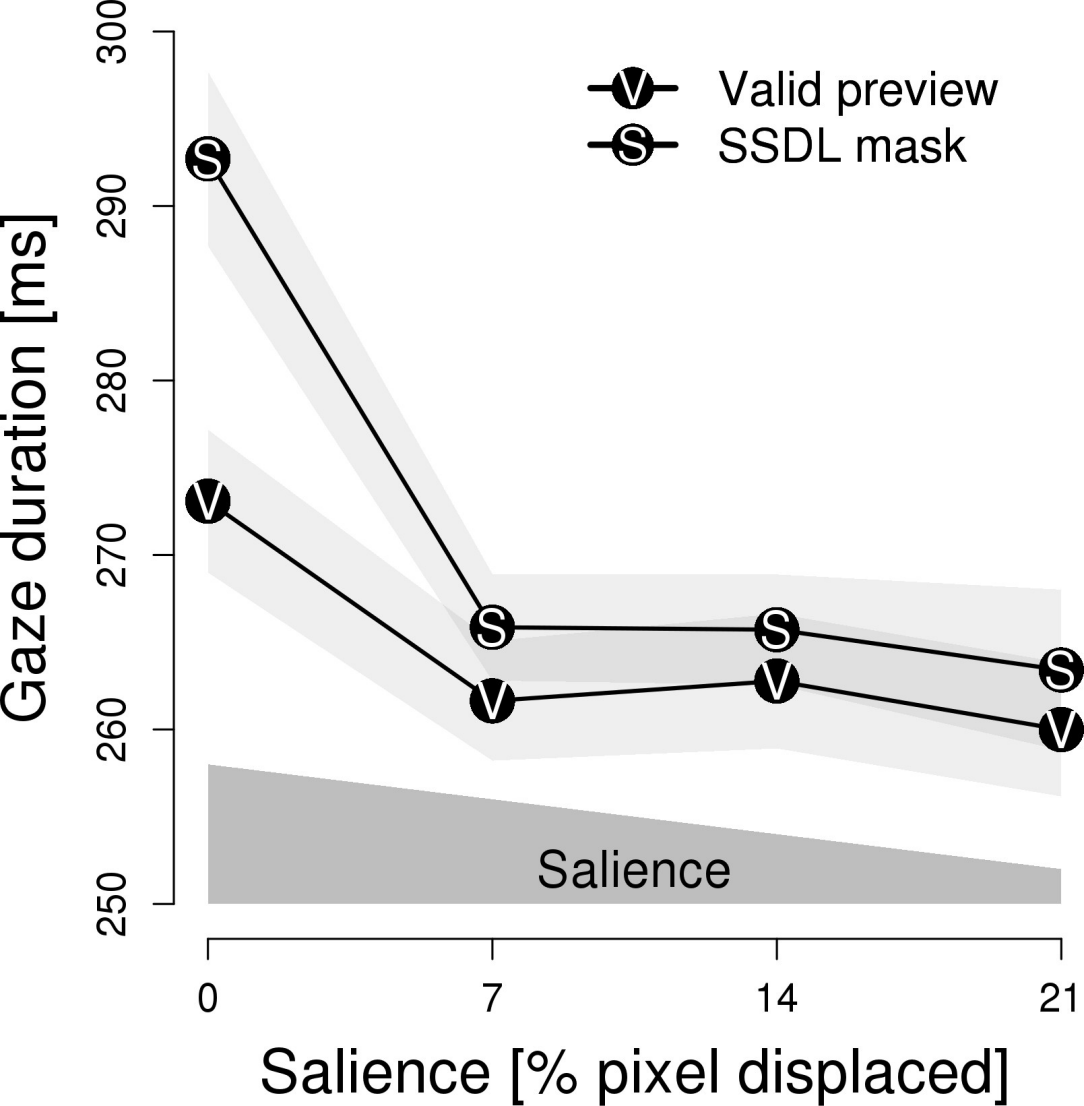
Experiment 4: Gaze duration on the pretarget word in relation to salience. Gaze durations on the pretarget word are depicted in relation to the salience of the parafoveal preview and are plotted separately for valid previews and same-shape, different letter masks. The light-gray areas represent 1 SEM. The polygon above the x-axis schematically indicates the level of salience.

The findings of the analysis of gaze duration on the pretarget word are presented in Fig 5. A two-way interaction of type of preview by salience; *F*(3, 90) = 2.77, *p* < .05, η = 0.004; qualified the significant main effects of type of preview; *F*(1, 30) = 4.51, *p* < .05, η = 0.004; and salience; *F*(3, 90) = 9.30, *p* < .001, η = 0.021. The theoretically relevant interaction was further investigated by separate analyses for masks and valid previews. For masks, a significant main effect of salience; *F*(3, 90) = 10.95, *p* < .001, η = 0.039; revealed that increasing perceptibility resulted in longer gaze durations (i.e., indicating preview costs). For valid previews, the main effect of salience was not significant, *F*(3, 90) = 2.31, *p* = .08.

#### Target words—The effects of visual degradation

**Fig 6:**
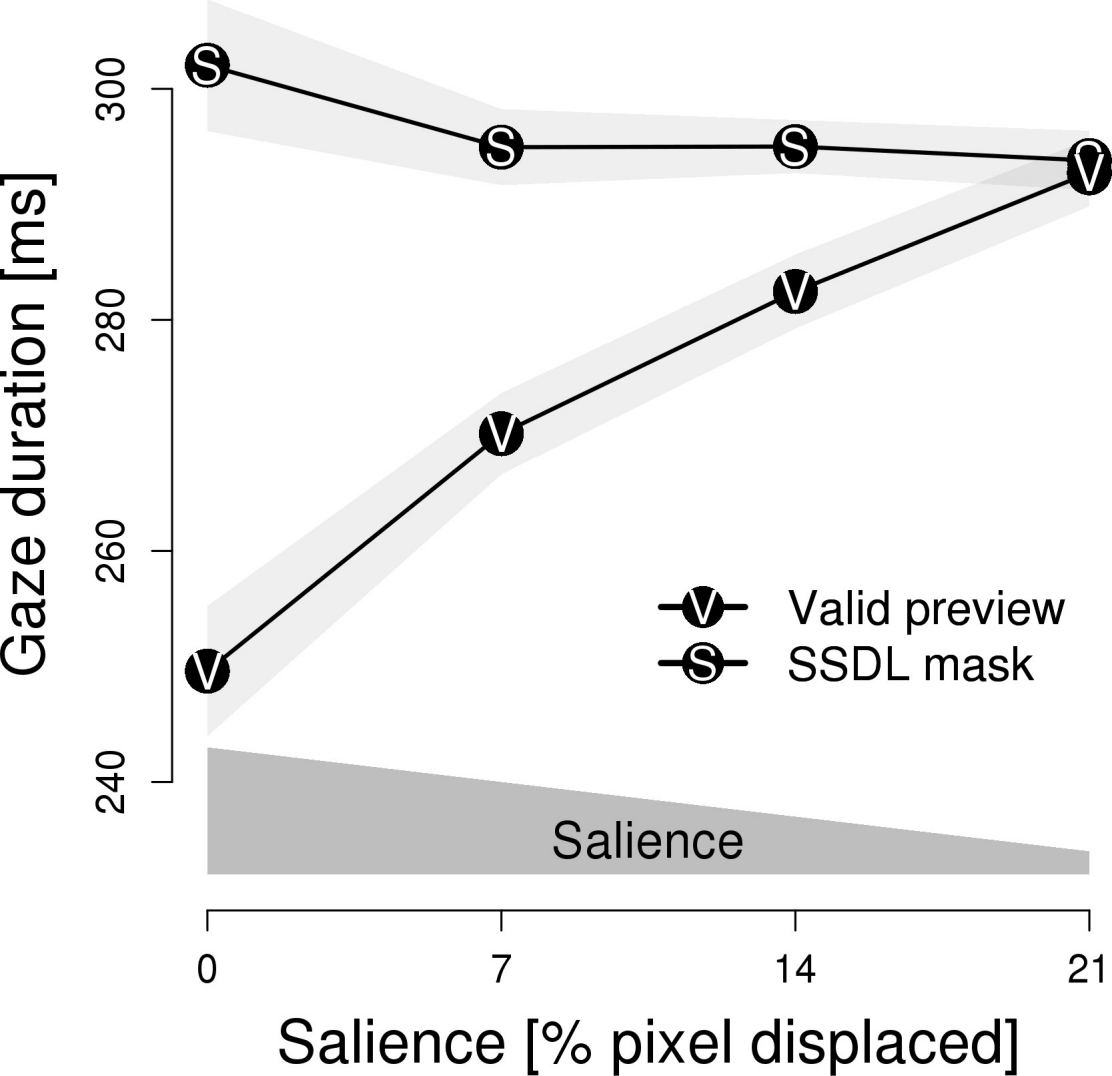
Experiment 4: Gaze durations on the target word in relation to salience. Gaze durations on the target word are depicted in relation to the salience of the parafoveal preview and are plotted separately for valid previews and same-shape, different letter masks. The light-gray areas represent 1 SEM. The polygon above the x-axis schematically indicates the level of salience.

The findings of the analysis of gaze duration on the target word are presented in Fig 6. An interaction of type of preview by salience; *F*(3, 90) = 17.23, *p* < .001, η = 0.030; qualified the main effects of type of preview; *F*(1, 30) = 26.74, *p* < .001, η = 0.041; and degradation; *F*(3, 90) = 9.33, *p* < .001, η = 0.014. The theoretically relevant, significant interaction was further investigated by separate analyses for masks and valid previews. For masks, the main effect of salience was not significant; *F* = 1.19. For valid previews, a significant main effect of salience; *F*(3, 90) = 23.71, *p* < .001, η = 0.097; revealed that increasing perceptibility resulted in shorter gaze durations (i.e., indicating preview benefits).

#### Target word—The effects of launch-site distance (i.e., salience-by-distance)

**Fig 7:**
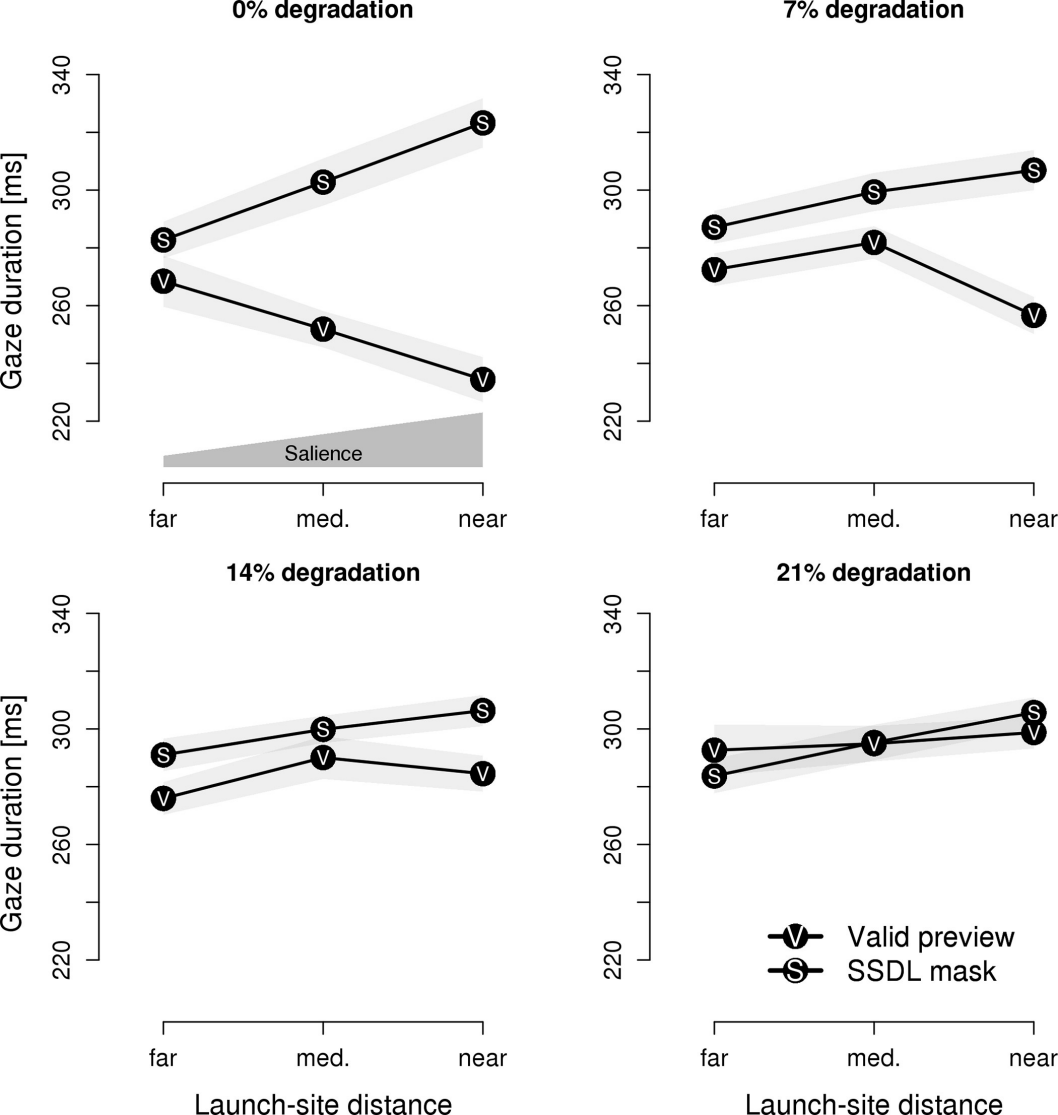
Experiment 4: Gaze durations on the target word in relation to launch-site distance. Gaze durations on the target word are depicted in relation to launch-site distance, with valid previews and same-shape, different letter (SSDL) masks plotted by separate lines. The different levels of visual degradation are plotted in separate panels. The light-gray areas represent 1SEM. The polygon above the x-axis (upper left panel) schematically indicates the level of salience.

The post-hoc analysis of launch-site distance (i.e., an analysis of salience-by-distance–comparable to that of [22]) is based on the natural variation in the length of the incoming saccades to the target words. To recapitulate, the closer the launch-site is to the parafoveal preview, the more salient was the preview during the preceding fixation. As near launch-sites we considered incoming saccades which originated from the ultimate or the penultimate character of the pretarget words (i.e., launch-site ≤ 2 characters including the space between the pretarget and the target word). As medium and far launch-sites, we considered launch-sites of 3 to 5 characters and 6 to 8 characters, respectively. The findings are depicted in Fig 7.

For the analysis, gaze durations on the target word were submitted to a 2 x 4 x 3 repeated measures ANOVA with type of preview (masks vs. valid preview), salience-by-perceptibility (0%, 7%, 14%, and 21% visual degradation) and launch-site distance (near, medium, far) as within-subject factors. The analysis revealed a two-way interaction of type of preview by launch-site distance; *F*(2, 60) = 21.83, *p* < .001, η = 0.012; which qualified the reliable main effects of type of preview; *F*(1, 30) = 27.99, *p* < .001, η = 0.035; and salience-by-perceptibility; *F*(3, 90) = 7.65, *p* < .001, η = 0.012. Further significant effects were the two-way interactions of type of preview by salience-by-perceptibility; *F*(3, 90) = 17.09, *p* < .001, η = 0.023; as well as the triple interaction between type of preview, salience-by-perceptibility and launch-site distance; *F*(6, 180) = 4.23, *p* < .001, η = 0.008. The salience-by-perceptibility by launch-site distance interaction was not significant; *F* < 1.

We followed up on the modulating effect of launch-site distance for the two different types of parafoveal previews with separate analyses for masks and valid previews. For masks, a reliable main effect of launch-site distance; *F*(2, 60) = 13.15, *p* < .001, η = 0.023; revealed that, as evident from Fig 7, closer launch-sites resulted in longer gaze durations on the target word–indicating preview costs. Neither the main effect of salience-by-perceptibility nor the interaction of salience-by-perceptibility with launch-site distance were significant, *F*s < 1. For valid previews, a salience-by-perceptibility by launch-site distance interaction; *F*(6, 180) = 3.52, *p* < .01, η = 0.018; qualified the main effect of salience-by-perceptibility; *F*(3, 90) = 19.70, *p* < .001, η = 0.071. Separate analyses for each level of degradation revealed that closer launch-sites resulted in shorter gaze durations (indicating a preview benefit) for undegraded previews; *F*(2, 60) = 7.37, *p* < .01, η = 0.059; and for previews degraded by 7%; *F*(2, 60) = 5.37, *p* < .01, η = 0.032; but not for previews degraded by 14% or 21%, *F*s < 1.15.

#### Target word—The effects of preview time (i.e., salience by exposure duration)

**Fig 8:**
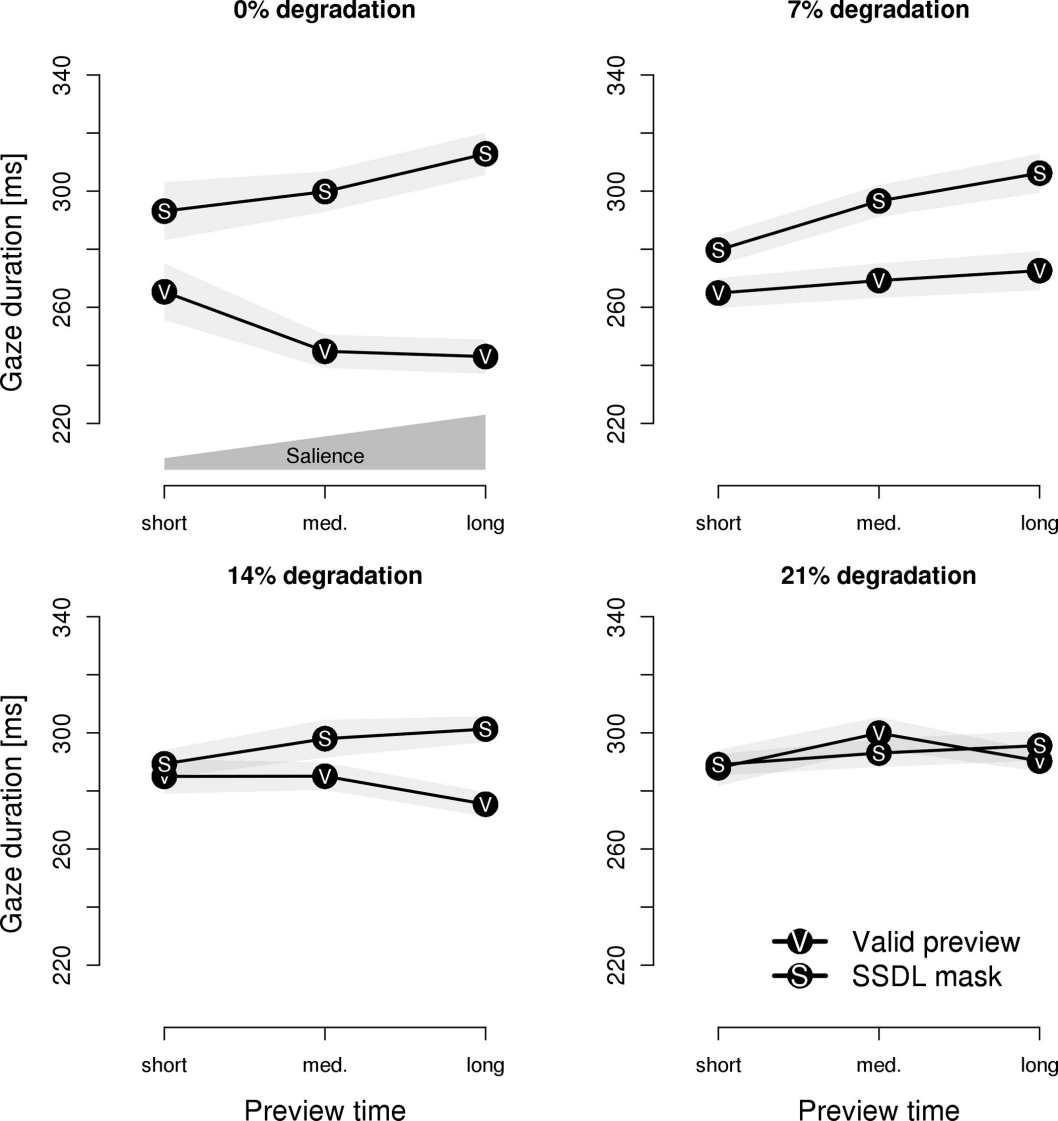
Experiment 4: Gaze durations on the target word in relation to preview time. Gaze durations on the target word are depicted in relation to preview time with valid previews and same-shape, different letter (SSDL) masks plotted by separate lines. The different levels of visual degradation are plotted in separate panels. The light-gray areas represent 1 SEM. The polygon above the x-axis (upper left panel) schematically indicates the level of salience.

The post-hoc analysis of preview time (*cf*. [22]) is based on the natural variation in the gaze durations on the pretarget word, allowing us to analyze the effect of the exposure time to the parafoveal previews. To operationalize preview time, gaze durations on the *pretarget* word were categorized on an individual basis for each participant as reflecting short exposures (i.e., gaze duration shorter than the individual 33th percentile), medium exposures (between the 33th and the 66th percentile) and long exposures (longer than the 66th percentile). The findings are depicted in Fig 8.

For analysis, gaze durations on the *target* word were submitted to a 2 x 3 x 4 repeated measure ANOVA with type of preview (masks vs. valid previews), exposure time (short, medium or long exposures) and saliency-by-perceptibility (0%, 7%, 14%, and 21% visual degradation) as within-subject factors. The analysis revealed reliable main effects of type of preview; *F*(1, 30) = 24.63, *p* < .001, η = 0.034; and saliency-by-perceptibility; *F*(3, 90) = 8.48, *p* < .001, η = 0.011. The main effect of preview time was not significant, *F*(2, 60) = 1.12. The main effects of type of preview and salience-by-perceptibility were qualified by two-way interactions of type of preview by salience-by-perceptibility; *F*(3, 90) = 15.02, *p* < .001, η = 0.024; and type of preview by exposure time; *F*(2, 60) = 14.03, *p* < .001, η = 0.005. The two-way interaction of salience-by-perceptibility by preview time as well as the triple interaction were not significant; *F*s < 1.65.

We followed up on the modulating effect of preview time for the two different types of parafoveal previews with separate analyses for masks and valid previews. For masks, a main effect of preview time; *F*(2, 60) = 6.37, *p* < .01, η = 0.010; revealed that increasing preview times resulted in longer gaze durations on the target word–indicating preview costs. Neither the main effect of salience-by-perceptibility nor the interaction were significant; *F*s < 1.32. For valid previews, a main effect of salience-by-perceptibility; *F*(3, 90) = 21.07, *p* < .001, η = 0.076; was qualified by the interaction of salience-by-perceptibility with preview time; *F*(6, 180) = 2.54, *p* < .05, η = 0.012. Separate analyses for each level of degradation revealed that for undegraded valid previews, increasing preview time resulted in shorter gaze durations–indicating a preview benefit; *F*(2, 60) = 4.98, *p* < .05, η = 0.037; whereas there was no modulatory effect of preview time for degraded stimuli; all *F*s < 1.60.

### Discussion

Experiment 4 investigated, whether parafoveal masks inflict comparable preview costs during sentence reading as we observed for the processing of isolated words. Furthermore, we assessed whether the benefits of valid previews are reliably captured by the incremental boundary paradigm.

#### Parafoveal masks inflict preview costs

Post-hoc analyses of launch-site distance and preview time revealed that parafoveal masks inflict preview costs during the processing of the target word. The closer the previous fixation (i.e., the launch-site of the incoming saccade) was to the parafoveally masked target word, the longer were the gaze durations on the target word. Likewise, the longer the readers remained on the pretarget word (i.e., the longer the exposure to the mask), the longer were the gaze durations on the target word. The processing costs on the target word indicated by launch-site distance and preview time, however, was not captured by the salience-by-perceptibility manipulation. It is important to note, however, that processing costs (which were indicated by launch-site distance and preview time; *cf*. [22]) are problematic when interpreting findings of the boundary paradigm—regardless whether the new incremental boundary paradigm is sensitive to these processing costs.

Concerning the *pretarget word*, the experiment revealed that increasing perceptibility of parafoveal masks resulted in prolonged gaze durations. This finding of a parafoveal-on-foveal effect of masks (but not for valid previews) indicates that the effects of masks are more extensive than simply inhibitory for the processing of the target word when it is eventually fixated. This finding may have implications for experiments on the effect of foveal load (see General Discussion). Furthermore, the (post-hoc) launch-site distance and preview-time analyses revealed that masks affected gaze durations on the target words for a broader range of saliences than valid previews.

Concerning the use of parafoveal masks during natural reading, the deciding question is, whether the findings of Experiment 4 suggest that masks inflict preview costs, but not whether (or to what extent) the novel incremental boundary paradigm captures these preview costs (since the novel technique is intended to assess benefits of valid previews, which do not necessarily go hand in hand with preview costs of masks). Summing up, the analysis of launch-site distance and preview-time for the target word and the analysis of salience for the pretarget word suggest substantial preview costs of parafoveal masks during sentence reading–corroborating the findings of [22].

#### The extent of the preview benefit

The incremental boundary paradigm reliably captured the *preview benefit* due to valid previews. Increasing perceptibility of valid previews resulted in shorter gaze durations on the target word. Manipulating the perceptibility of valid previews, however, did not affect fixation times on the pretarget word. Experiment 3 revealed that a degradation by ~20% results in a zero-information condition which does not provide any preview benefit. In Experiment 4, a comparison of processing times between undegraded previews and previews that were degraded by 21% allows us to estimate the extent of the preview benefit on the target word which is 45 ms. Interestingly, our estimate is comparable to the preview benefit of 30–50 ms reported for adult English readers [9] that should be subject to overestimation. Concerning the present experimental setting, we can only speculate to what extent a classical boundary paradigm would have overestimated the preview benefit. Analysis of visual salience suggest an overestimation of 10 ms, whereas an analysis of launch site distance suggests an overestimation of 40 ms–resulting in an (over-)estimated preview benefit between 55 ms and 85 ms. Although our estimates exceed those reported for English readers, they are comparable to the values we reported in a recent study with German-reading children [23].

## General discussion

Recent studies [21, 22, 23] indicated that parafoveal masks inflict preview costs and thus are not a suitable baseline condition in studies on parafoveal preprocessing. The present study presented the *incremental boundary paradigm* (a fusion of the boundary paradigm and the incremental priming technique; [2] and [26], respectively). The objectives were to further investigate the nature of parafoveal masks and to assess the feasibility of the incremental boundary paradigm for the study of parafoveal preprocessing.

### Parafoveal masks inflict preview costs

The present findings corroborate the existing evidence that parafoveal masks inflict preview costs. These costs were evident for the processing of isolated words (Experiments 1 and 2) as well as during natural reading (Experiment 4). The manipulation of salience-by-distance (Experiment 1) and the post-hoc analysis of launch-site distance and exposure duration (Experiment 4) revealed that increasing salience of parafoveal masks resulted in prolonged processing times on the target word. This finding was substantiated by the experiments using the salience-by-perceptibility manipulation (Experiments 2 & 4). Moreover, Experiment 4 revealed that parafoveal masks also interfere with the processing of the *pretarget* word (see [32] for a similar finding).

### Overestimation of preview benefit–misinterpretation of preview costs

When used in classical boundary experiments, the (hidden) preview costs of parafoveal masks may result in an overestimation of the preview benefit. When we compare processing times during an experimental condition which presents (partially) valid previews to those during the allegedly neutral baseline condition (i.e., parafoveally masked previews), we would misinterpret the prolonged processing in the baseline condition (which is in fact inflicted by the parafoveal masks) as facilitated processing in the experimental condition. The consequence would be an *overestimation of the preview benefit*. The observed difference in processing times between the two conditions might reflect both preview costs inflicted by the masks in the baseline condition and preview benefits of the experimental condition. In the worst-case, however, an (factually) ineffectual experimental condition might be *misinterpreted* as providing a preview benefit.

Parafoveal masks not only result in an overestimation of the preview benefit. Rather, the extent to which we observe preview costs is modulated by the way in which salience is manipulated (salience-by-perceptibility, saliency-by-distance, salience-by-preview-time) and whether we assess processing on the pretarget word or target word. Furthermore, these factors seem to interact. To illustrate, for valid previews, the effects of launch-site distance and preview time were modulated by the salience-by-perceptibility manipulation, whereas no such a modulatory effect was observed for parafoveal masks (*cf*. Fig 7 and Fig 8). Furthermore, during sentence reading salience-by-perceptibility did not reveal preview costs of parafoveal masks on the target word (but did so during the recognition of isolated words), whereas launch-site distance and preview time did reveal preview costs. Trying to control for these modulating factors (let alone, controlling for the interaction of the factors) would be very difficult to accomplish.

### Alternative masks vs. visual degradation

Given the finding that SSDL masks inflict processing costs, one might wonder whether a different kind of parafoveal mask (in combination with the boundary paradigm) might be more suitable for the study of parafoveal preprocessing. Concerning X-masks, we could show [21] that these masks inflict preview costs as well. It seems that any “orthographic” deviation from a valid preview may inflict preview costs. The reason why visually degraded previews, even if degraded beyond the point of zero-information, do *not* inflict preview costs might be that visually degraded previews do not provide orthographically aberrating information.

### Parafoveal masks are only a means to an end

From a theoretical perspective, we are interested in the parafoveal processing of *valid* previews. Parafoveal masks are only a means to an end for investigating the effect of valid previews in the context of natural reading (in order to realize a baseline condition—as discussed in detail in the Introduction, parafoveal masks need to be differentiated from manipulations of parafoveal preview used, e.g., to study semantic preview benefits. The latter approach is not subject to overestimations). In this light, parafoveal masks have been instrumental for the study of parafoveal preprocessing, but are (for several research questions) no necessity–making possible to use the novel incremental boundary paradigm when suitable.

The foremost advantage of the incremental boundary paradigm is that it allows investigators an interpretation along the rationale of a within-condition baseline–which renders an explicit, external baseline condition unnecessary. Rather, the direction of the effect of a parafoveal preview (facilitatory or inhibitory) can be inferred from the effect of the experimentally manipulated salience of the preview. Faster processing due to increasing salience is indicative for a preview *benefit*. Slower processing due to increasing salience is indicative for preview *costs*. These interpretations do not necessitate anchoring by means of an “external” baseline condition.

Moreover, a level of salience that provides zero parafoveal information allows researchers to estimate the absolute size of the preview benefit (cf. [26]). In the present study, visual degradation of around 20% was sufficient to annihilate the extraction of information from the parafovea. An orthogonal combination of the manipulation of eccentricity and salience in Experiment 3 revealed that visual degradation of parafoveal previews does not result in preview costs, but reduces the availability of parafoveal information to zero. As a consequence, a degradation by 20% provides a within-condition baseline that allows investigators to estimate the absolute size of preview benefits (or costs; but see Footnote 1).

### Consequences and future perspectives

While further research is necessary to corroborate the processing costs of parafoveal masks as indicated by the present and previous studies [21, 22, 23], the potentially erroneous interpretations of findings from the classical boundary paradigm might put into perspective a large body of existing evidence. It might be necessary to re-investigate those studies which presupposed that parafoveal masks are neutral. In the following, we will exemplarily address two such research topics in more detail, namely the studies on i.) parafoveal-on-foveal effects, the effect of foveal load and the spillover effect and ii.) parafoveal preprocessing in dyslexic and beginning readers.

#### Parafoveal-on-foveal, foveal load and spillover Effects

The present findings are relevant with respect to studies which investigate the spillover effect or the effect of foveal load. These studies investigate, whether the processing demands of the currently fixated word modulate the extent to which an upcoming target word is preprocessed (i.e., the foveal load effect; [33]) or whether the processing demands of the pretarget word are propagated to the target word (i.e., the spillover effect; e.g., [34]). In these studies, the processing demand of the pretarget word is manipulated (e.g., by manipulating its frequency) while either a masked preview (assumed to constitute a zero-information preview) or a valid-preview is presented parafoveally. Warren and colleagues [35] argued that an artificial interaction of processing demands and the parafoveal preprocessing of masks might jeopardize the interpretation of this experimental approach.

While we could show that parafoveal masks inflict preview costs, we can not judge whether the extent of these preview costs are modulated by the processing demands of the pretarget word. It could be assumed, however, that a more narrow attentional window (caused by increasing processing demands) might modulate (similar to, e.g., the effect of launch-site distance) the extent of the preview costs of parafoveal masks. Moreover, in Experiment 4 (*cf*. Fig 5) we examined parafoveal-on-foveal effects and found that parafoveal masks (but not valid previews) resulted in prolonged fixation times on the *pretarget word* (see also [32]). Thus, masks inflicted parafoveal-on-foveal processing costs. As yet, we do not know to which extent these parafoveal-on-foveal costs affect the subsequent processing of the target word. For the studies on the effect of foveal load or the spillover effect, it is impossible to resolve these artificial effects of parafoveal masks post-hoc–not least since we do not know yet how these effects interact.

In a recent study, we investigated the foveal load effect and the spillover effect in beginning readers with the incremental boundary paradigm [36]. The study did not reveal an effect of foveal load, but a substantial spillover effect. Another recent study [37] re-assessed the effect of foveal load and only obtained a pattern of findings indicative of the effect when they applied parafoveal letter masks, but not for various other preview manipulations. This finding suggests that the ostensible effect of foveal load is indeed elicited by previewing orthographically illegal letter strings (but see [32]). Using the incremental boundary paradigm would not only help to avoid artificial interactions [35], but it would also avoid the overestimation of the preview benefit [23].

#### Parafoveal preprocessing in beginning and dyslexic readers

In Experiment 4, the effect of preview time indicated that the preview costs inflicted by parafoveal masks are more pronounced, the longer the reader fixate the pretarget word. An open question, however, is whether such an effect also holds true for readers that generally exhibit longer fixation times–such as non-proficient (e.g., beginning or dyslexic) readers. Using a salience-by-perceptibility manipulation, [23] could show that children exhibit substantial preview costs for parafoveal SSDL masks (as well as for parafoveal X-masks). Thus, beginning readers may exhibit more pronounced preview costs for parafoveal masks than proficient, adult readers.

If preview costs are modulated by reading proficiency, then the estimation of the preview benefit in non-proficient readers is especially prone to overestimations. In fact, in [23] we could show that preview benefits, if estimated by means of the classical boundary paradigm with parafoveal masks, would have been overestimated by almost 50%. In dyslexic readers, a modulatory effect of reading proficiency might question the evidence on their perceptual span and their amount of parafoveal preprocessing.

### Conclusion

The present findings and the available evidence [21, 22, 23] question the validity of some of the conclusions drawn on the basis of the classical boundary paradigm in combination with parafoveal masks. Consequently, findings from studies that use parafoveal masks as baseline condition need to be interpreted with reservation. The novel incremental boundary paradigm is an alternative experimental approach that renders the use of parafoveal masks unnecessary.

## References

[1] McConkie GW, Rayner K. The span of the effective stimulus during a fixation in reading. Percept Psychophys. 1975; 17: 578–586.

[2] Rayner K. The perceptual span and peripheral cues in reading. Cognit Psychol. 1975;7: 65–81.

[3] Shillcock R, Roberts M, Kreiner H, Obregón M. Binocular foveation in reading. Atten Percept Psychophys. 2010;72: 2184–2203. 10.3758/APP.72.8.2184 21097862

[4] Kirkby J, Blythe H, Drieghe D, Benson V, Liversedge S. Investigating eye movement acquisition and analysis technologies as a causal factor in differential prevalence of crossed and uncrossed fixation disparity during reading and dot scanning. Behav Res. 2013;45: 664–678. 10.3758/s13428-012-0301-2 23344736

[5] Rayner K. Eye movements and the perceptual span in beginning and skilled readers. J Exp Child Psychol. 1986;41: 211–236. 3701249 10.1016/0022-0965(86)90037-8

[6] Hohenstein S, Laubrock J, Kliegl R. Semantic Preview Benefit in Eye Movements During Reading. J Exp Psychol Learn Mem Cogn. 2010;36: 1150–1170. 10.1037/a0020233 20804291

[7] Hohenstein S, Kliegl R. Semantic preview benefit during reading. J Exp Psychol Learn Mem Cogn. 2014;40: 166–190. 10.1037/a0033670 23895448

[8] Veldre A, Andrews S. Is semantic preview benefit due to relatedness or plausibility? J Exp Psychol Hum Percept Perform. 2016;42: 939–952. 10.1037/xhp0000200 26752734

[9] Rayner K. Eye movements and attention in reading, scene perception, and visual search. Q J Exp Psychol. 2009;62: 1457–1506. 10.1080/17470210902816461 19449261

[10] Briihl D, Inhoff AW. Integrating Information Across Fixations During Reading. J Exp Psychol Learn Mem Cogn. 1995;21: 55–67. 10.1037/0278-7393.21.1.55

[11] Gagl B, Hawelka S, Richlan F, Schuster S, Hutzler F. Parafoveal preprocessing in reading revisited: evidence from a novel preview manipulation. J Exp Psychol Learn Mem Cogn. 2014;40: 588–595. 10.1037/a0034408 24041397

[12] Inhoff AW. Parafoveal Processing of Words and Saccade Computation During Eye Fixations in Reading. J Exp Psychol Hum Percept Perform. 1989;15: 544–555. 10.1037/0096-1523.15.3.544 2527961

[13] Johnson RL, Perea M, Rayner K. Transposed-Letter Effects in Reading. J Exp Psychol Hum Percept Perform. 2007;33: 209–229. 10.1037/0096-1523.33.1.209 17311489

[14] Lima SD, Inhoff AW. Lexical Access During Eye Fixations in Reading. J Exp Psychol Hum Percept Perform. 1985;11: 272–285. 10.1037/0096-1523.11.3.272 3159838

[15] Rayner K, Well AD, Pollatsek A, Bertera JH. The availability of useful information to the right of fixation in reading. Percept Psychophys. 1982;31: 537–550. 10.3758/BF03204186 7122189

[16] Veldre A, Andrews S. Parafoveal preview benefit is modulated by the precision of skilled readers' lexical representations. J Exp Psychol Hum Percept Perform. 2015;41: 219–232. 10.1037/xhp0000017 25384238

[17] Rayner K, Slowiaczek ML. Expectations and parafoveal information in reading: Comments on McClelland and O'Regan. J Exp Psychol Hum Percept Perform. 1981;7: 645–651. 10.1037/0096-1523.7.3.645

[18] Rayner K, McConkie GW, Ehrlich S. Eye movements and integrating information across fixations. J Exp Psychol Hum Percept Perform. 1978;4: 529–544. 10.1037/0096-1523.4.4.529 722245

[19] McClelland JL, O'Regan JK. Expectations increase the benefit derived from parafoveal visual information in reading words aloud. J Exp Psychol Hum Percept Perform. 1981;7: 634–644. 10.1037/0096-1523.7.3.634

[20] McClelland JL, O'Regan JK. On visual and contextual factors in reading: A reply to Rayner and Slowiaczek. J Exp Psychol Hum Percept Perform. 1981;7: 652–657. 10.1037/0096-1523.7.3.652

[21] Hutzler F, Fuchs I, Gagl B, Schuster S, Richlan F, Braun M, et al Parafoveal X-masks interfere with foveal word recognition: evidence from fixation-related brain potentials. Front Syst Neurosci. 2013;7: 33 10.3389/fnsys.2013.00033 23888130 PMC3719217

[22] Kliegl R, Hohenstein S, Yan M, McDonald SA. How preview space/time translates into preview cost/benefit for fixation durations during reading. Q J Exp Psychol. 2013;66: 581–600. 10.1080/17470218.2012.658073 22515948

[23] Marx C, Hawelka S, Schuster S, Hutzler F. An incremental boundary study on parafoveal preprocessing in children reading aloud: Parafoveal masks overestimate the preview benefit. Cogn Psychol. 2015;27: 549–561. 10.1080/20445911.2015.1008494 26246890 PMC4487581

[24] Jordan TR, Thomas SM, Patching GR. Assessing the Importance of Letter Pairs in Reading-Parafoveal Processing Is Not the Only View. J Exp Psychol Learn Mem Cogn. 2003;29: 900–903. 10.1037/0278-7393.29.5.900 14516223

[25] Jonides J. & Mack R. On the cost and benefit of cost and benefit. Psychol Bull. 1984;96: 29–44.

[26] Jacobs AM, Grainger J, Ferrand L. The Incremental Priming Technique: A Method for Determining Within-Condition Priming Effects. Percept Psychophys. 1995;57: 1101–1110. 10.3758/BF03208367 8539086

[27] McDonald SA. Effects of number-of-letters on eye movements during reading are independent from effects of spatial word length. Vis cogn. 2006;13: 89–98. 10.1080/13506280500143367

[28] Hutzler F, Braun M, Võ ML, Engl V, Hofmann M, Dambacher M, et al Welcome to the real world: Validating fixation-related brain potentials for ecologically valid settings. Brain Res. 2007;1172: 124–129. 10.1016/j.brainres.2007.07.025 17803976

[29] Raaijmakers JGW, Schrijnemakers JMC, Gremmen F. How to Deal with "The Language-as-Fixed-Effect Fallacy": Common Misconceptions and Alternative Solutions. J Mem Lang. 1999;41: 416–426.

[30] Clark HH. The language-as-fixed-effect fallacy: A critique of language statistics in psychological research. Journal of Verbal Learning and Verbal Behavior. 1973;12: 335–359. 10.1016/S0022-5371(73)80014-3

[31] Ziegler JC, Ferrand L, Jacobs AM, Rey A, Grainger J. Visual and phonological codes in letter and word recognition: evidence from incremental priming. Q J Exp Psychol. 2000;53: 671–692. 10.1080/027249800410508 10994225

[32] Findelsberger E., Hutzler F., Hawelka S. Spill the load: Mixed evidence for a foveal load effect, reliable evidence for a spillover effect in eye movement control during reading. Atten Percept Psychophys. In press. 10.3758/s13414-019-01689-5 PMC6647363 30843176

[33] Henderson JM, Ferreira F. Effects of Foveal Processing Difficulty on the Perceptual Span in Reading—Implications for Attention and Eye-Movement Control. J Exp Psychol Learn Mem Cogn. 1990;16: 417–429. 2140401 10.1037//0278-7393.16.3.417

[34] Risse S, Kliegl R. Evidence for delayed parafoveal-on-foveal effects from word n+2 in reading. J Exp Psychol Hum Percept Perform. 2012;38: 1026–1042. 10.1037/a0027735 22428669

[35] Warren T, Reichle ED, Patson ND. Lexical and Post-Lexical Complexity Effects on Eye Movements in Reading. J Eye Mov Res. 2011;4: 1–10. 21603125 PMC3097123

[36] Marx C, Hawelka S, Schuster S, Hutzler F. Foveal processing difficulty does not affect parafoveal preprocessing in young readers. Sci Rep. 2017;7: 41602 10.1038/srep41602 28139718 PMC5282480

[37] Veldre A., Andrews S. How does foveal processing difficulty affect parafoveal processing during reading? J Mem Lang. 2018;103: 74–90. 10.1016/j.jml.2018.08.001

